# A multiperspective investigation of the underrepresentation of minoritized ethnic participants in dementia research and proposed strategies to improve inclusive recruitment practices

**DOI:** 10.1002/alz.70129

**Published:** 2025-04-06

**Authors:** Natalie L. Marchant, Elahi Hossain, Shiping Chen, Geeti Kabra, Laiba Ahmad, Ione Fraser, Lorraine Cezair‐Phillip, Aikaterini Fotoupoulou, Harpreet Sihre, Naaheed Mukadam, Anna L. Cox

**Affiliations:** ^1^ Division of Psychiatry University College London London UK; ^2^ UCL Interaction Centre University College London London UK; ^3^ Member of the public; ^4^ Department of Clinical Educational & Health Psychology University College London London UK; ^5^ ARC North Thames Academy Primary Care and Population Health UCL Medical School (Royal Free Campus) University College London London UK; ^6^ Camden and Islington NHS Foundation Trust, St. Pancras Hospital London UK

**Keywords:** Alzheimer's disease, diversity, ethnic minorities, ethnicity, race, underrepresented, underserved

## Abstract

**INTRODUCTION:**

Minoritized ethnic populations are underrepresented in dementia research despite having differing risk and prognosis profiles. We sought to gain multiperspective insights about why minoritized ethnic groups are underrepresented in dementia research.

**METHODS:**

Qualitative data were collected from focus groups with individuals from minoritized ethnic backgrounds in the UK (*n* = 13) and open‐ended survey responses from international dementia researchers (*n* = 54). Facilitators and barriers to the inclusion of minoritized ethnic populations were examined using the COM‐B model of behavior.

**RESULTS:**

Community member barriers included lacking knowledge about dementia and research opportunities, language, stigma, and medical/institutional racism. Facilitators included using varied recruitment strategies, culturally appropriate language, communal research, and salient outcomes. Researcher barriers included lacking skills, knowledge, materials, time, and funding to sustainably engage with minoritized ethnic communities. Facilitators included culturally sensitive, community‐centered approaches in diverse research groups.

**DISCUSSION:**

Our study contributes multiperspective insights about the underrepresentation of minoritized ethnic populations in dementia research and proposes strategies to improve inclusive research practices.

**Highlights:**

- Minoritized ethnic communities are underrepresented in dementia research.
- Community members want varied, culturally tailored recruitment strategies and communal research with salient outcomes.
- Researchers describe lacking skills, time, and funding for sustainable engagement.
- Targeted recruitment campaigns and inclusive research programs are recommended.

## 1 INTRODUCTION/BACKGROUND

Dementia is a public health priority, affecting more than 55 million people worldwide, with rising prevalence.^1^ Despite significant advances,^2^ a cure or route to prevention has remained elusive. In high‐income Western countries, dementia disproportionately affects people from minoritized ethnic backgrounds,^3^, ^4^ yet the underrepresentation of ethnically minoritized participants in research has severely impeded a comprehensive understanding of dementia, quality of healthcare provision, and development of effective treatments.^5^, ^6^

In this article, we use “ethnicity” as it refers to clusters of people who have common geographical and/or ancestral cultural traits.^7^ While “minority” refers to people in the minority of their country of habitation, we use “minoritized” to emphasize the active processes of marginalization or oppression faced by certain minority ethnic groups.^8^ We avoid using “race” because it is a social construct used to categorize people based on visible physical characteristics and behavior.^9^ However “racism” exists due to the pervasive repercussions of the historical use of racial categorization to falsely assert the biological hierarchy of racial groups.^7^

Within the UK where dementia is the leading cause of death, minoritized ethnic groups have a 20% higher risk of dementia, younger age of diagnosis, and die earlier after diagnosis compared with ethnically White groups,^10^ yet they are critically underrepresented in research. The most recent systematic review of randomized controlled trials of treatments to improve dementia revealed that only 39.4% of trials reported the ethnicity of participants with a pooled proportion of 11.4% of participants coming from non‐White backgrounds.^11^ Of trials conducted in the UK, only one reported ethnicity with 5.1% of participants included from minoritized ethnic backgrounds.^3^ This underrepresentation spans different research domains.^4^ For example, the two largest publicly available dementia neuroimaging datasets – the National Alzheimer's Coordinating Center (NACC, USA) and the UK Biobank (UK) – comprise 83.3% and 95.5% White participants, respectively,^12^ despite the fact that most recent census data record 75.7% White (USA) and 81.7% White (England and Wales) populations. Studies need to have a sufficiently large number of ethnically diverse participants to detect potential differences and improve the generalizability of results^13^, ^14^; therefore examination of factors that affect the inclusion of ethnically minoritized groups in dementia research is needed.

Brijnath et al.^15^ conducted a scoping review to examine approaches researchers have taken to enhance the recruitment of minoritized ethnic groups in published dementia studies. Most articles came from the USA (86%), with two from the UK^16^, ^17^ (3%). Language adaptations were the most reported approach, followed by engaging in community outreach and providing flexible ways to participate. Indeed, major initiatives in the USA incorporating these approaches to recruit underrepresented groups in dementia research are beginning to yield positive results (e.g., Rivera Mindt et al.^18^).

Within the UK, Waheed and colleagues^19^ conducted a systematic review to identify published challenges faced by dementia researchers in the recruitment of minoritized ethnic communities. Six themes that hindered recruitment emerged: community attitudes and beliefs about dementia (e.g., stigma), the recruitment process (e.g., underrepresentation in health services, lack of/misdiagnoses), data collection issues (e.g., language, literacy, cultural understanding), practical issues (e.g., financial resources, time requirements), researcher characteristics (e.g., discrepancies in ethnicity and age between researchers and participants), and lack of normative data.

Much work has emerged from the USA elucidating factors that affect participation in dementia research from perspectives of minoritized ethnic populations within that country (e.g., Black Americans).^20^, ^21^ Within the UK, studies have examined perceptions of dementia and, for example, memory problems,^22^ service use,^23^, ^24^ care,^25^ and help seeking,^26^, ^27^, among minoritized ethnic communities; however, first‐hand questioning of community members about their perceived facilitators and barriers to participating in dementia research has been lacking. Listening to these communities is essential to inform inclusive recruitment strategies.

This study involved directly asking individuals from Black and South Asian minoritized ethnic communities in the UK, given their underrepresentation in research and documented differences in dementia risk and prognosis,^3^, ^10^ about their perceptions of research, how they would like to be approached and engaged in research, and barriers and facilitators to participation. Further, because published reports may not always include recruitment approaches, facilitators, and barriers, we also conducted a global survey of dementia researchers about these issues. While general guidance exists to support the recruitment of underrepresented populations in research (e.g., TRIAL FORGE^28^), specificities associated with dementia (e.g., age, cognitive impairment, stigma) call for the investigation of facilitators and barriers to participation that may be particular to this condition. Our objective was to gain multiperspective insights about why minoritized ethnic groups are underrepresented in dementia research and generate strategies to improve inclusion.

## 2 METHODS

To gain a multiperspective understanding of why there is an underrepresentation of minoritized ethnic populations in dementia research, we conducted a qualitative study composed of focus groups and an online survey that adhered to consolidated criteria for reporting qualitative studies (COREQ)^29^ (Appendix A). The focus groups aimed to uncover the behavioral barriers and facilitators to minoritized ethnic populations living within the UK participating in dementia research, while the global online survey aimed to uncover the facilitators/barriers to dementia and aging researchers recruiting minoritized ethnic populations into dementia research.

### 2.1 Diversity, equity, and inclusion statement

Researchers worked collaboratively with community partners during the design and execution of the study to ensure the centrality of diversity, equity and inclusion. Community partners advised on recruitment materials (e.g., to help ensure inclusive language and format). Participants in the focus groups were offered to join an online or in‐person focus group during different times of day (including evening), based on participant preferences and needs to accommodate caring responsibilities, mobility, work schedules, digital literacy, and so forth. We provided support to participants to complete online forms and to join the online focus groups, when needed. Researchers and community partners co‐designed the topic guides and co‐led the focus groups to help ensure inclusive language was used and to minimize perceptions of hierarchy. Community partners were compensated for their time, in line with National Institute for Health and Care Research / Innovations in Clinical Trial Design and Delivery for the Under‐served (NIHR/INCLUDE) guidelines.

**RESEARCH IN CONTEXT**

1. **Systematic review**: We reviewed previously published literature about the inclusion of minoritized ethnic groups in dementia research using PubMed. Previous work regarding recruitment strategies to include these groups was largely country‐specific, predominantly emerging from the USA, and none included perspectives of minoritized ethnic community members from the UK.
2. **Interpretation**: This qualitative study adds to the current literature by using a behaviorally informed approach to provide a multiperspective understanding from minoritized ethnic community members and dementia researchers of facilitators and barriers regarding the underrepresentation of minoritized ethnic participants in dementia research, with particular focus on the UK and Europe. Strategies to improve inclusive participant recruitment are also proposed.
3. **Future directions**: It is hoped that the adoption of the proposed strategies will improve inclusive research practices. Increased inclusion of ethnically minoritized participants will contribute to a comprehensive understanding of dementia and dementia risk, quality of healthcare provision, and development and testing of effective medications or interventions in diverse populations.

### 2.2 Participants

#### 2.2.1 Focus group

To be eligible for the focus group, participants needed to be aged 18 or older, identify as being from a Black or South Asian background (given their documented elevated risk of dementia^3^), have fluency in the English language, know a close friend or relative with dementia, or be living with early dementia themselves if they had the capacity to consent and attend the group with a close friend, family member, or carer. We purposively sought to include participants who had not previously participated in research.

Participants were recruited through opportunistic and snowball sampling via community partners, community groups, and charities who support people from minoritized ethnic backgrounds and/or people affected by dementia via phone, text, and email. Participants from previous research projects who consented to be contacted about future research studies were also contacted via phone.

#### 2.2.2 Online survey

To be eligible for the online survey, participants needed to conduct research with human participants in an aging and/or dementia‐related research area.

Survey participants were recruited through colleagues and collaborators via email, professional networks, including the Alzheimer's Association International Society to Advance Alzheimer's Research and Treatment (ISTAART) professional organization newsletter (a global network of scientists, clinicians, and dementia professionals), and advertisement on X (formerly Twitter).

### 2.3 Materials

#### 2.3.1 Focus group guide

The focus group topic guide was co‐developed by researchers and community partners (N.L.M., I.F., L.C.‐P., and H.S.). The guide explored the following topics: their understanding of “research” and awareness of taking part in research, how they would like to hear about research participation opportunities, what would encourage them to participate, facilitators and barriers to taking part in research, and how researchers could engage with communities such as theirs to improve recruitment strategies (see Appendix B).

#### 2.3.2 Online survey

The survey was developed by N.L.M., N.M., and A.C. and hosted on the online Research Data Collection Service (REDCap) web platform. The survey collected demographic information (age, gender, ethnicity, ethnic background), academic position, research domain, and an open‐ended response section that asked participants about their current recruitment practices and facilitators and barriers to recruiting minoritized ethnic populations (Appendix C).

### 2.4 Procedure

The study was registered with the University's Data Protection Officer and received ethics approval (8503/003 and UCLIC_2024_001_Cox_Marchant). All participants provided written informed consent before attending the focus group or completing the online survey.

Focus groups were held online via the Zoom platform and conducted by a researcher and community partner (N.L.M. and I.F.) using a semi‐structured schedule. N.L.M., the researcher, is a non‐clinical (PhD) Associate Professor in the Division of Psychiatry at University College London, has been a family carer of someone with dementia, and is a woman of mixed Black‐American/White‐British background. I.F., the community partner, has extensive experience facilitating groups, has been a family carer of someone with dementia, and is a woman of mixed Black‐Caribbean/White‐British background. All participants were screened over the phone by N.L.M. or I.F. and provided sociodemographic information using the online REDCap web platform, with assistance from a member of the research team if needed, before joining a focus group. Each focus group session began with introductions of the facilitators (including the researcher's personal goals and reasons for conducting the research), the purpose of the focus groups, and reminders about the voluntary nature of participation, confidentiality, and that the session would be recorded. A brief presentation about dementia was followed by discussions facilitated by the community partner using the topic guide. Each focus group lasted approximately 90 min, and participants were remunerated £37.50 ($48.30). The recorded sessions, instead of field notes, were transcribed using the automated audio Zoom transcription service, which was manually checked by L.A. and G.K. They were not returned to participants for comment or corrections.

Participants in the online survey first provided sociodemographic and professional information and then completed the open‐ended response questions. The survey took 5 to 15 min to complete. Participants were not given financial remuneration but were provided the option to have their name and affiliation added to the list of contributors to the manuscript.

Collected data (focus group transcripts, open‐ended survey responses) were then securely stored in University College London's Data Safe Haven for analysis.

### 2.5 Data analysis

#### 2.5.1 COM‐B model of behavior

The COM‐B model of behavior was used as the guiding framework to systematically explore the behavioral reasons for the current underrepresentation of minoritized ethnic populations in dementia research. The model posits that for any behavior to occur, there must be sufficient capability (C), opportunity (O), and motivation (M).^30^, ^31^ These components are each heuristically divided into two types:

- Capability refers to either an individual's *physical capability* (having the necessary physical skills, strength, or stamina) or *psychological capability* (having the necessary knowledge, psychological skills, strength, or stamina) to perform a behavior.
- Opportunity refers to external influences on behavior that fall under either *physical opportunity* (environmental affordances or barriers in terms of time, resources, locations, and cues) or *social opportunity* (interpersonal influences, social cues, and cultural norms).
- Motivation refers to internal processes that energize and direct behavior which fall under either *reflective motivation* (self‐conscious plans and evaluations: beliefs of what is good and bad) or *automatic motivation* (emotional reactions, desires: wants and needs, impulses, and reflex responses).

The COM‐B model components were used to identify the barriers and facilitators to our target behaviors and, in turn, what components need to change for potential intervention strategies to be effective. Our mutual target behaviors of interest in this study were the following: (1) minoritized ethnic populations participating in dementia research and (2) researchers recruiting minoritized ethnic populations in dementia research.

The COM‐B model was chosen as the leading framework for the analysis for several reasons:

1. The model provides a well‐organized and comprehensive overview of the possible internal and external influences on behavior – striking a healthy balance between not being too simple (e.g., the EAST framework^32^) and too complex in its explanation (e.g., the Behavioral Drivers Model^33^).
2. The model is well suited to be applied within a public health context, with existing examples of it being used to specifically explore individuals' participation in health research.^34^, ^35^, ^36^
3. The model in practice aligns well with the qualitative research methods utilized in this study – with multiple existing examples of it being used to analyze qualitative data from focus groups (e.g., Timlin et al.^37^ ^)^ and surveys (e.g., Johnson et al.^34^).

#### 2.5.2 Approach

Focus group transcripts and open‐ended survey responses were imported into NVivo 12 and analyzed separately using reflexive thematic analysis.^38^ Our analyses adopted the following theoretical position: an *essentialist* and *experiential* orientation to the data (we assumed a unidirectional relation between language and meaning and that expressed thoughts, feelings, and experiences reflect internally held states), a *semantic* coding of the data (i.e., codes aimed to reflect the surface meanings of data), and a blend of both *inductive* and *deductive* orientation to the coding of data (described in the next paragraph). We chose not to utilize the concept of data saturation (i.e., the point at which no new thematic insights can emerge from data) to guide our data collection and analysis. This is because, according to Braun and Clarke,^39^ the concept of data saturation does not align with the fundamental values and assumptions of reflexive thematic analysis. Rather than holding the assumption that meaning resides in the data waiting to be *discovered *by the researcher, reflexive thematic analysis advocates that meaning is *constructed *through a situated and reflexive interpretative process, making it difficult to align the method with notions of saturation – as new meaning is therefore always theoretically possible. Deciding when to end data collection and analysis was therefore not dictated by data saturation, but rather – following Braun and Clarke's recommendation – was achieved through an ongoing in situ interpretative judgment by E.H. that considered the *adequacy* (richness, complexity) of the data for addressing the research question.

The analyses involved EH first conducting the familiarization stage, which involved reading the transcripts in full and making initial notes and observations. Then the initial coding involved a blend of both a deductive and inductive orientation. The deductive analysis involved using components from the COM‐B model of behavior (Capability: Psychological, Physical, Opportunity: Physical, Social, and Motivation: Automatic, Reflective) as a top‐down sensitizing concept through which to explore and make sense of the data, which involved coding data extracts to the relevant COM‐B components. This was followed by an inductive analysis to identify more precise data‐specific themes, which involved openly coding the COM‐B organized data extracts to capture their semantic meaning. Themes were then generated in an iterative process whereby codes were examined, merged/renamed where necessary, and organized hierarchically within the wider COM‐B framework. The final themes were then reviewed, defined, and named. Participants did not provide feedback on the themes.

To ensure the quality of the analyses of both focus group transcripts and open‐ended survey responses (i.e., a rigorous, systematic, and reflexive analysis was conducted), EH cross‐checked the analytical approach taken against Braun and Clarke's “15‐point quality checklist for thematic analysis.”^38^ Reflexive thematic analysis moves away from notions of truth existing “out there” to be uncovered by the researcher, rather positing that qualitative insights are generated through an active and reflexive analytic process. The checklist contains criteria that span the reflexive process: transcription (e.g., “the data have been transcribed to an appropriate level of detail”), coding and theme development (e.g., “each data item has been given thorough and repeated attention in the coding process”), analysis and interpretation (e.g., “analysis and data match each other – the extracts evidence the analytic claims”), overall analysis (e.g., “enough time has been allocated to complete all phases of the analysis adequately”), and the written report (e.g., “the specific approach to thematic analysis, and the particulars of the approach, including theoretical positions and assumptions, are explicated”). Furthermore, we also utilized *triangulation* as a quality‐checking strategy. This is because our study is a multiperspective exploration of the underrepresentation of minoritized ethnic populations in dementia research, affording us a richer, more multifaceted understanding of the topic.

## 3 RESULTS

We present the results of the focus groups and then the online survey. For each, we describe the barriers and facilitators to capabilities, opportunities, and motivations to participating in dementia research and recruiting minoritized ethnic populations, respectively. Coding trees for focus groups and the researcher survey are in Appendix D.

### 3.1 Focus groups: barriers/facilitators to minoritized ethnic populations participating in dementia research

All participants who completed the screening agreed to participate in the study; however, one participant dropped out before joining the focus group due to a scheduling conflict. Three focus group sessions, each with three to six participants, were conducted with a total of 13 participants (Table 1). Focus groups were not intentionally separated by ethnicity, age, diagnosis of dementia, or any other variable; however, by chance, two groups comprised participants from Black ethnic backgrounds, and one comprised participants from South Asian ethnic backgrounds.

**TABLE 1:** Focus group participant characteristics.

Participant	Age	Gender	Ethnicity or ethnic background (self‐reported)	Previously participated in research	Diagnosis of dementia
F1P1	40 to 44	Woman	Black – Caribbean or Caribbean British	Yes	No
F1P2	50 to 54	Woman	Black – Other Background	No	No
F1P3	35 to 39	Woman	Mixed or multiple ethnic groups – White and Black Caribbean	No	No
F1P4	55 to 59	Woman	Black – Caribbean or Caribbean British	No	No
F1P5	50 to 54	Man	Black – Caribbean or Caribbean British	No	No
F2P2	30 to 34	Woman	Black – Caribbean or Caribbean British	No	No
F2P3	40 to44	Woman	Black – Caribbean or Caribbean British	No	No
F2P4	65 and over	Woman	Black – Caribbean or Caribbean British	Yes	No
F2P5	60 to 64	Woman	Black – Caribbean or Caribbean British	No	No
F2P6	60 to 64	Woman	Black – Caribbean or Caribbean British	Yes	No
F3P2	65 and over	Man	Asian – Bangladeshi or Bangladeshi British	Yes	Yes
F3P3	60 to 64	Woman	Asian – Indian or Indian British	Yes	No
F3P4	65 and over	Woman	Asian – Bangladeshi or Bangladeshi British	Yes	No

#### 3.1.1 Psychological capability

##### Lack of knowledge about mental health issues and research (barrier)

Minoritized ethnic participants showed a lack of knowledge about mental health disorders (e.g., dementia). This lack of knowledge acted as a primary barrier to understanding what dementia research entails in the first place and why it is important for them to participate in it:

*“I think that the understanding about the actual disease itself is what you're saying is also something that inhibits us from getting involved in the research. Is that it? Am I… Am I right in saying that?” (Facilitator)*

*“That's what I'm saying. [A] lack of understanding.” (P4, F1)*

##### Lack of knowledge about dementia research participation opportunities (barrier)

Participants showed a lack of knowledge of what channels to look for to participate in dementia research – with the assumed routes for participation, for example, general practitioners (GPs)/health services seemingly not offering these opportunities. They also showed a lack of understanding of what participating in such research would entail which made them hesitant to engage with the topic – suggesting a need for better communication about the realities of what dementia research participation involves:

*“But in terms of, yeah, engaging in studies, it's just where do we find out that information? Where's it advertised? Is it advertised on, you know, platforms that people would not ordinarily look at…?” (P3, F1)*

*“The other is about having as much knowledge and information as possible. How intrusive is this research going to be?… knowledge is really good, that would make me participate. Full knowledge and the knowledge of services that are out there, and other research that's going on, that I could perhaps be involved with and talk nonstop.” (P2, F1)*

*“But we, as Black people, are never told by GPs … or never told by hospitals …” (P3, F3)*

##### Older populations lack the technological skills to access online‐only services (barrier)

Older participants lacked the technological skills to find out about opportunities to participate in mental health research. This skill deficit means that intervention strategies should consider other offline channels to engage with this specific population.

*“If you've got older people that participate, they may not use social media and things like that. So how else can you target these people and let them know that these studies are happening. Cause they're really important.” (P3, F1)*

##### Language barriers limit the ability to participate in dementia research (barrier)

Participants acknowledged that language barriers were a key barrier for minoritized ethnic populations to participate in dementia research. This impacted the population's ability to gain knowledge about mental health issues and to participate in the research itself.

*“…because, particularly South Asian communities, Bengali, communities, Pakistani and other communities, they have a language barrier. And if that barrier can be resolved and if it is publicized, you know, all over all the community, I think we can prevent some of these diseases.” (P3, F3)*

#### 3.1.2 Physical opportunity

##### Time availability to participate in dementia research (barrier)

Participants noted time as a barrier to participating in dementia research. There was uncertainty of how much time it would take to participate in research, and – in the context of more group‐based research – finding a time and place that suits everyone.

*“That's exactly what I wanted to say, the timing, because to do a research [study], you have to give in a lot of time and not just getting the information, but it's collating the information.” (P5, F1)*

##### Offline targeted dementia research recruitment strategies (facilitator)

Participants highlighted the need for dementia research to promote participation opportunities through offline means that are explicitly targeted at the communities of interest. This involves promoting in offline spaces that minoritized ethnic populations would be interacting in (e.g., public transport/events, medical spaces, religious venues, minoritized ethnic group‐specific organizations) and through offline materials (e.g., leaflets, newspapers). This is especially important to make it more accessible for less technically proficient – often older – populations.

*“I worked with a local Caribbean association where I live … But what I learned by working with this Caribbean Association is that there are Black representatives in unions. If, you know, if you wanted to get people in the workplace and catch things early … in councils … So probably places where I'd access in my community. In addition to GP practices and carer services.” (P2, F1)*

*“Because in East London, for example, [it] would be a good area to have these advertisements up where you have, in terms of the demographic, you have people of colour. And I think targeting a certain cultural background in order to get the information. So, if this is done whether on public, in public transports, on trains, on buses, I think it would be a good avenue.” (P4, F1)*

*“…or maybe like a coffee morning, like every fortnight, where you can check in and not just physically be on social media, because some people are not very techy or savvy. Some people like to sit down and have a coffee and interact, and, you know, have that communication with a group setting and stuff like that. So, I'd say that kind of method.” (P3, F2)*

##### Online and media‐based targeted dementia research recruitment strategies (facilitator)

Participants highlighted the need for dementia research recruitment to also occur through digital means (e.g., online questionnaires/sign‐up lists, content on social media platforms) and more conventional broadcast media (e.g., TV shows, adverts). Such strategies would address the identified lack of knowledge about mental health issues, with such campaigns possibly promoting greater education and awareness raising about these conditions:

*“Getting the information out there. I think it is very important to have maybe a social media. Yeah, social media. What would you say? … “stage” in which to advertise this and to say, you know, people have TikTok and maybe get it out there so people can know that it's real. It's happening. And if you see these signs what to do, what actions to take.” (P4, F1)*

*“And I was thinking, as you were saying, that is, is things like, actually something that is very general. And that's TV. Most of us watch TV. Most of us watch news. Most of us will watch adverts. I know that this costs an absolute fortune. But actually, even if it's something about advertising where we can go to get involved in studies and research, that is, is of any interest to us, not just around dementia and stuff. But actually, you know, having a little exposé about those kinds of things. Cause actually, I think those things are really, really important.” (P1, F1)*

#### 3.1.3 Social opportunity

##### Social stigma attached to mental illness within the community (barrier)

Participants pointed to social stigmatization (and general apathy) of mental illness/dementia within their communities as a strong factor in not engaging with the topic in general. Individuals with dementia are often looked down upon (e.g., seen as foolish), and mental health/illness research is not very valued, indicating that these social norms are underpinned by a lack of knowledge/understanding about mental health/illness. These cultural norms act as a barrier to engaging with the topic – linking to the barrier of negative emotions (e.g., fear):

*“…In my experience I find that people that are going through dementia, other people look at them like they've turned foolish and what they say is no longer as applicable as it used to be.” (P6, F2)*

*“…in terms of dementia, like what would've been said before around people accepting – especially from our background – mental health … the stigma that's attached to it.” (P3, F1)*

*“Well amongst the Africans, you know, it seems as if when you're told there's an indication that somebody has Alzheimer's … just words that have gone ‘round … myths, and so on, that, you know, this person is not there? Because you have this condition, you say it's seen as a disease, not a condition of any sort. And I think this is part of lack of knowledge” (P4, F2)*

##### Institutional racism (barrier)

Participants pointed out how institutional racism (within health services, education, and local authorities) and linked inequalities (e.g., educational gaps/opportunities) negatively impacted the ability of minoritized ethnic populations to engage in dementia research, whether that means having the necessary access to medical services that could/or should direct them to such opportunities, the general attitude of local authorities of minoritized ethnic populations prevents conversations opening up about increasing accessibility to such opportunities or being in an educational position to know about such research. This barrier relates to psychological skill deficits (e.g., knowledge, language) acting as a barrier to participating in research. Participants pointed out that the onus should not necessarily be on the minoritized ethnic populations in developing these skills to engage in this research, but rather on the institutions reaching out and having the capacity to engage with these populations as they are right now, for example, non‐English speakers. Further, the experience of institutional racism also links to negative emotional barriers (described in the following section), as the constant toll of the experience will further reduce the motivation to try and participate in such research:

*“And you know it's an ongoing battle that we have to face, of challenging things that should be given to us naturally, and that actually wears you down over time and means that you're not necessarily gonna seek out other opportunities because actually, we're exhausted with doing what we just need to do to survive.” (P1, F3)*

*“… a lot of our communities don't understand what dementia is. They don't understand the signs and symptoms* *. They call to the doctors and the doctors usually just feed them back … ‘It's stress’ or ‘it's mental health’ or whatever, and they don't get the right diagnosis until very, very late. And then by that time there's no prevention that's suitable for them. So that's where daily mortality happens. And I think that's an inequality issue. And that's an institutional racism issue.” (P3, F3)*

##### Trustworthy personalities in the promotion and running of dementia research (facilitator)

The inclusion of trustworthy individuals from similar minoritized ethnic backgrounds in the promotion (e.g., in TV shows/adverts) and mediation (i.e., in focus groups) of their participation in dementia research is an important social facilitator. Participants outlined aspects such as expertise and being from the same cultural background as important factors that in turn can help to build trust. COVID champions were highlighted as a useful model that could be adopted by dementia research recruitment planners to follow as a way to spread awareness about research opportunities and encourage the participation of minoritized ethnic individuals in dementia research.

*“So, the point I'm making is that you know it. It could be through an interview. It could be where, you know, someone goes on and is interviewed about dementia, about how it impacts on families, you know, of Black African, Caribbean and Asian origin, and then put the call out for people to participate in research and guaranteed people like my mother will be knocking on the door.” (P2, F1)*

*“I'm thinking about having people that are representative. So, making sure it is somebody of colour or someone that you can relate to. So, they're asking you those vulnerable questions you feel that you already have something in common, and so it builds trust to start with.” (P3, F1)*

*“…during Covid there was a group of people meeting one of them that are deemed as a Covid champion! I believe that something like that might be necessary, as you know, with the champions you were bringing from outside what is happening, what the thoughts are, what is going to help people do that because we're talking about, you know, having these groups and expertise, and so on. But I don't believe that you're going to get anywhere if … If you don't have people who are champions…” (P4, F2)*

##### Culturally sensitive recruitment communication strategies (facilitator)

Participants emphasized the importance of the language used in future research recruitment materials. They highlighted aspects such as considering the tone and being culturally aware/sensitive to ensure the message is received in the right way. This links to the idea of social/cultural stigmas around mental illness within minoritized ethnic groups and that ensuring the language used in any strategies is sensitive to these issues:

*“And I think this is a big thing. Language is a very, very big thing, especially with us from the ethnic minorities. We have to overcome the culture. Yeah, of either somebody from East Africa to somebody from the West Indies, or somebody from up North, somebody from South. Do you know what I mean?” (P5, F1)*

##### Dementia research that is communal in nature (facilitator)

Participants highlighted the importance of dementia research being done in a bottom‐up, community‐driven way, for example, group meetings. They expressed wanting to engage in research that connects them to others with the same lived experiences – citing the very focus groups they were participating in as an ideal model for how such research and knowledge sharing should be conducted. Elements of shared meaning‐making, camaraderie, and support are central to how participants wanted dementia research to be conducted (whether in terms of recruitment or the actual data collection approach). By engaging the community, spreading awareness and talking about dementia and its research, and participating in research in a public and communal way, participants felt it could help to normalize the topic and challenge culturally embedded stigmas/preconceptions – thereby reducing this social barrier.

*“I would want to know that I'm being connected with people who are experiencing a similar [thing]. I wouldn't say the same because I think it's different for all of us based on our backgrounds and circumstances, but similar…” (P2, F1)*

*“I think it's for people to know from each other people that there are other people [who] are going through the same exact thing, and it is not something to be ashamed of, but it's something to talk about, and the more we talk about it, the more it becomes almost natural and know that this is not what you would call a taboo or something.” (P4, F1)*

*“Oh, also these sorts of sessions [focus group], you know, would help. I mean, you know, as you have started with this sort of session, and this sort of session will be of help for people who would like to know about all these things.” (P4, F3)*

#### 3.1.4 Automatic motivation

##### Negative emotions linked to lived experience (barrier)

Participants described a range of negative emotions that acted as barriers to participating in dementia research such as fear, anxiety, shame, loneliness (due to not wanting to be a burden on others), and anger. These emotions were attributed to several interacting causes: (1) the lived experience of dementia, (2) medical institutional experiences or past medical racism, (3) assumptions/lack of knowledge about dementia research, and (4) social stigma about mental illness held within the community. This general fear highlights the importance of dementia research being conducted openly and communally where participants can feel it is a safe space to express their experience.

*“With watching my mom, I'll see how she sort of just went so quiet like [when] friends came round. She wouldn't participate. She didn't think she was worthy, you know … whatever she says didn't matter … They sort of go into their shell you know … So, I would say, fear, pride losing them, feeling they're not worthy.” (P5, F2)*

*“I've heard the word ‘research.’ For some reason I have a negative connotation with it. And I think that just that that little, those little few seconds of having that negativity stems from our history as a people of Black Africans, and I'm mixed, I'm South Asian and African Caribbean, but connotations, negative connotations due to the history of treatment and research that was carried out on Black people, gynecologically and the syphilis episode. So I get a little bit nervous when I hear the word ‘research.’ And every time I hear about research being carried into certain things that apply to me, and I think ‘oh, I could put myself forward, or perhaps Dad could, Me and Dad could participate in this.’ There's a bit of me that holds back because that comes to mind, and it makes me nervous, and I imagine it may be similar for other people too.” (P2, F1)*

#### 3.1.5 Reflective motivation

##### Seeing the practical consequences of participating in dementia research for oneself, family, and the wider community (facilitator)

Participants felt they would be motivated to participate in dementia research if they were informed of and saw the real‐world/practical consequences to themselves, family, and the wider community of participating in it:

*“…it's good to take part in research. But you want to hear results coming from that …” (P5, F1)*

*“… knowing that it's not going to be used against you. It'll be used for a purpose to make change in life, you know, in the long run, is to identify how we with our experiences can help somebody else out there can help others who are going through the same thing.” (P4, F1)*

*“You want to give information, but you know that the information you're giving is going to cause changes, not just to my life, but to other people's life.” (P5, F1)*

### 3.2 Online survey: barriers/facilitators to researchers recruiting minoritized ethnic populations into dementia research

In total, 54 aging and dementia researcher participants were recruited (Table 2).

**TABLE 2:** Online survey participant characteristics.

Variable	Count (*n* = 54)	Percentage (%)
**Age Range**		
25 to 29	4	7
30 to 34	9	17
35 to 39	6	11
40 to 44	11	20
45 to 49	10	19
50 to 54	7	13
55 to 59	3	6
60 to 64	1	2
65 and over	3	6
**Gender**		
Man	16	30
Woman	37	69
Non‐binary	1	2
**Minoritized ethnic background**		
No	44	81
Yes	9	17
Prefer not to say	1	2
**Career stage**		
Graduate/postgraduate student	8	15
Postdoctoral	5	9
Early career	11	20
Mid‐career	15	28
Senior	13	24
Other	2	4
**Continent**		
Asia	3	6
Europe	42	78
North America	4	7
South America	1	2
Oceania	4	7

#### 3.2.1 Psychological capability

##### Perception of minoritized ethnic populations lacking certain skills or knowledge (barrier)

Researchers pointed out how minoritized ethnic populations lacked certain skills and knowledge (e.g., linguistic, educational level, health literacy, technology literacy) to include them in their dementia research. Low fluency in the local language is often an exclusion criterion to research, and researchers also highlighted how previous research experience with minoritized ethnic populations indicated that these populations lacked knowledge of mental health issues and how to participate in dementia research – confirming findings from focus groups.

*“I have just run an event with local community stakeholders for Afro‐Caribbean communities, the biggest barriers identified were lack of awareness of these opportunities and lack of awareness of dementia and brain health within the communities generally.” (SP1)*

*“Limited English fluency is an exclusion criterion for most of our research studies on older adults. This may potentially exclude some older adults who do not feel that their level of English is fluent.” (SP36)*

##### Researchers lack the skills and knowledge to engage with minoritized ethnic populations (barrier)

Researchers highlighted how they lack the necessary knowledge and skills to conduct research with minoritized ethnic populations. Specifically, they highlighted problems in how to reach these populations during study recruitment, as well as language barriers (e.g., translating) once they have recruited from such populations. This highlights the need to include researchers from minoritized ethnic populations to facilitate this dementia research and training for researchers on engaging in more inclusive research:

*“Lack of awareness for me as a researcher about inclusive recruitment practices (where to go to find ethnically diverse participants).” (SP32)*

*“Language barriers can hinder communication and understanding, making it hard to recruit or explain the purpose of the study.” (SP45)*

#### 3.2.2 Physical opportunity

##### Difficulty recruiting minoritized ethnic populations through default channels and methods (barrier)

Researchers found recruiting minoritized ethnic populations through their usual recruitment channels (e.g., clinical databases, hospitals) and strategies (e.g., snowballing) to be ineffective:

*“Additionally, snowball and convenience sampling are most prevalent in my field, and these practices rely on those already engaged in services that are easily accessed.” (SP42)*

*“Lack of outreach in diverse communities further exacerbates this issue, as traditional recruitment methods might not reach these groups.” (SP45)*

##### Minoritized ethnic populations accessing the research site (barrier)

Researchers pointed out that minoritized ethnic populations faced problems traveling to research sites to participate due to temporal and financial barriers (i.e., time to get there/cost to travel), confirming focus group perceptions. Researchers noted how conducting research (when possible) more locally in venues they know, for example, community centers, sports clubs, and religious venues, and through online (e.g., remote phone calls) means would be beneficial in terms of increasing recruitment and would align with conducting dementia research that is more communal in nature:

*“There can also be barriers related to procedures when there is a lack of flexibility on how and where data is collected. Requiring people to come to a research center or hospital in working hours is a barrier for many people.” (SP2)*

*“… we have tried to undertake more of our research activities in the community setting e.g. in community centres, sports clubs, Mosque/faith centres which has been more acceptable and favoured” (SP33)*

*“Allowing remote/phone participation assisted a lot with recruitment in my study.” (SP45)*

##### Culturally diverse dementia research public engagement (facilitator)

Researchers were aware of the importance of engaging in culturally diverse public engagement around dementia research (e.g., campaigns, and science communication). They outlined how implementing targeted advertising strategies/science communication through relevant channels to minoritized ethnic populations could increase their participation. Importantly, this facilitator requires specialized funding/projects, which is currently a salient barrier:

*“To help recruit more ethnically/racially diverse participants, we need to be able to target our recruitment advertisements at more diverse populations, accompanied by wider engagement with diverse community groups (e.g., via research promotion talks, rather than advertisements alone).” (SP36)*

*“Disseminating information through trusted platforms like radio stations or community newsletters.” (SP45)*

##### Lack of financial funding to sustainably engage with minoritized ethnic communities (barrier)

Researchers pointed out that a lack of financial resources was a key barrier to recruiting minoritized ethnic populations. Money was required to develop community partnerships (necessary to build trust), hire researchers with expertise engaging with minoritized ethnic populations, pay participants for participation and travel costs/hire local venues to come to them, adapt research materials to be culturally compliant (e.g., translation costs), and implement recruitment strategies.

*“We need: Money from funders towards more research assistants speaking different languages to help recruit in local communities – money to cover participation fee and travel costs for participants – money to cover a venue for research within the local community – money towards translating materials and/or recording videos that can be used in recruitment.” (SP5)*

*“Money: To pay for representatives of ethnically diverse communities to write/review/refine study documents and processes and be involved as PPI [patient public involvement] at all stages. Money: To pay for meetings/presentations with gatekeepers of those communities. Money: To produce reports appropriate for the needs of different communities.” (SP26)*

*“Funding. In order to recruit, we need to develop trustworthy and long‐standing collaborations and relationships.” (SP51)*

##### Lack of research materials that can be used by minoritized ethnic populations (barrier)

Researchers highlighted how not having research materials required for recruiting a minoritized ethnic population was a barrier. For example, a lack of materials (e.g., instruments) that have been translated to the population language (which links to researcher language skill deficit) or access to assessment tools that are sensitive to cultural diversity:

*“Most assessment tools and outcomes (e.g., cognitive measures) are not sufficiently sensitive to cultural diversity.” (SP30)*

*“Provision of culturally appropriate artifacts.” (SP52)*

##### Research delivery time pressures (barrier)

Researchers were aware that they had time pressure to recruit and run their research (e.g., working to grant deadlines), which conflicted with the time required to engage properly with minoritized ethnic populations (i.e., to build the necessary trust, relationships/connections/network).

*“Time pressure to recruit means that we feel pressure to recruit the most accessible participants and so ambitions to maximise diversity in recruitment end up sidelined.” (SP32)*

*“Trusting relationships take time to develop with minoritised communities.” (SP14)*

*“These practices take time, and when working to a grant deadline, all but the most crucial work seems to be deprioritised.” (SP42)*

#### 3.2.3 Social opportunity

##### Healthcare systems not identifying and meeting the needs of diverse populations (barrier)

Researchers were aware of how systemic issues within healthcare systems could lead to problems in reaching minoritized ethnic populations, for example, clinicians needing to actively target these populations and address accessibility to dementia services. This supports focus group perceptions of ethnicity‐based health inequalities within medical/health institutions acting as a barrier:

*“Lack of diversity in clinical research due to the healthcare system failing to identify and meet the needs of those from diverse populations.” (SP29)*

*“In the clinic I work in, there is not much diversity on the caseload itself, possibly reflecting access issues with that clinic.” (SP38)*

*“More openness from clinicians and from clinics to actively target recruitment towards more diverse groups.” (SP9)*

##### Community‐centered dementia research approaches (facilitator)

Researchers highlighted the importance of engaging in bottom‐up community‐centered dementia research as a possible way to increase the recruitment of minoritized ethnic populations. This supports perceptions from the focus group of dementia research being more communal in nature. Researchers outlined the importance of including minoritized ethnic populations within the whole research cycle in an inclusive way: meeting participants where they live, building strong relationships with local contacts/organizations embedded within minoritized ethnic communities (which can even be facilitated through wider national charities/organizations), co‐producing research projects (i.e., objectives and aims) that are relevant to the community needs and subsequent materials, and sharing findings with these communities:

*“Research development that starts by engaging the communities to learn about their concerns.” (SP2)*

*“Co‐production of all study material to make sure it is fit for purpose and attractive to as many as possible.” (SP25)*

*“Engagement work with communities prior to starting research to establish trust, and to consider what research approaches will work best or where approaches will need adaption/a complete rethink to better meet the needs of the potential participant population; ongoing engagement with relevant community groups and encouragement of feedback through the research project so that issues can be addressed as the research progresses with adaptions made as necessary; ongoing relationships with communities following research including feedback on obtained results and next steps, and ideally providing a gain for the community (e.g., in our own research we have provided seminars to the community on what dementia is and provided an opportunity for communities to be able to ask questions etc.” (SP35)*

##### Greater valuation of inclusive dementia research practices within wider research culture (facilitator)

Researchers pointed out a need for dementia research culture (e.g., journals, grant funders) to better value (and thus incentivize) research that recruits minoritized ethnic populations. This links to funding and time pressure issues that researchers currently face in recruiting minoritized ethnic populations:

*“Journals and funders need to insist that all publications on projects include data on diversity.” (SP5)*

*“Funders could set recruitment targets with respect to diversity and acknowledge the additional time that it may take to build relationships with diverse communities.” (SP29)*

##### Low cultural diversity in research groups (barrier)

Researchers pointed out that there is a lack of (and need for greater) cultural diversity within their research teams. Greater team diversity would help to address the lack of current researchers’ skills and knowledge to engage with minoritized ethnic populations (e.g., language abilities, connections to these communities) and identify unforeseen barriers to recruiting within minoritized ethnic populations and may help to increase the value of more inclusive dementia research practices:

*“It helps to have a diverse research team speaking different languages and representing different communities you want to collaborate with – it helps to recruit in the local communities – it helps to give back to communities, e.g., by providing education on a topic or some other service.” (SP5)*

*“Underrepresentation of these populations among employees at our university is also problematic.” (SP44)*

#### 3.2.4 Reflective motivation

##### The perception that minoritized ethnic populations are skeptical about participating in dementia research (barrier)

Researchers were aware that minoritized ethnic populations held negative evaluations (i.e., being skeptical/wary/untrusting/disinterested) about participating in dementia research due to various reasons (e.g., cultural, bad past experiences, medical racism). They understood that to change this, the onus was on them as researchers to build trust with these populations and that making it clear how the research can benefit community members was key to facilitating their engagement. This corroborates the negative emotional barriers and need for salient consequences outlined within the focus groups:

*“Scepticism towards participating in research is much greater amongst non‐European individuals we approach with our studies.” (SP16)*

*“The feeling of being ‘used’ or ‘hard to reach’ among these communities – they are not hard to reach and are very willing to be a part of research and want to, but often past experiences have tainted their trust for the process. Researchers have taken what they needed and not returned with findings, dissemination information or stayed in contact which has been a negative experience.” (SP33)*

*“Gaining access to communities and community liaisons takes time and building these relationships requires trust if you are not a part of this community already.” (SP31)*

## 4 DISCUSSION

We conducted a multiperspective study using the COM‐B model of behavior to understand the current underrepresentation of minoritized ethnic populations in dementia research. To achieve this, we implemented focus groups with minoritized ethnic community members within the UK and a global online survey with dementia researchers that explored the facilitators/barriers to participating in dementia research and recruiting minoritized ethnic populations, respectively. This qualitative study adds to the current literature base by taking a direct first‐hand and behaviorally informed approach to understanding the facilitators and barriers to participating in dementia research.

The study findings revealed that knowledge and skills were key barriers to target within the psychological capability subcomponent of COM‐B. Minoritized ethnic populations lacked knowledge about mental health/illness issues and routes to participate in dementia research, language fluency to currently participate, and technological skills – particularly older populations – to discover participation opportunities online. Additionally, dementia researchers lack knowledge of how to reach minoritized ethnic populations and language skills to practically engage them in research, supporting prior findings of the importance of providing cultural competence training to researchers to promote Alzheimer's research participation in Black Americans.^20^

The importance of deploying targeted offline and online/media‐based recruitment strategies as opposed to using default channels/methods was identified as key within the physical opportunity subcomponent. Time and distance to research sites were also key physical opportunity barriers – supporting prior findings on the importance of greater flexibility in when, where, and how dementia research takes place to support Alzheimer's disease research participation among Black Americans.^21^ Further, researchers lacking funding, adapted research materials, and salient research time pressures were key physical opportunity barriers to sustainably recruiting minoritized ethnic populations.

Social stigma around mental health/illness, systemic racism in healthcare, and the importance of recruitment strategies and dementia research practices being culturally and communally orientated (i.e., conducting community‐based participatory research) were identified as key within the social opportunity subcomponent, supporting prior findings on the importance of dementia research involving long‐term sustainable relationships with minoritized ethnic communities to promote Alzheimer's disease research participation.^20^ Further, the lack of cultural diversity within dementia research groups and the need for a research culture to more highly value inclusive research practices were also key social opportunity issues – with the former insight supported by prior findings that dementia research should ensure clinical trial staff teams reflect the minoritized ethnic demographics being served.^20^

Within motivation, there were key issues within both the automatic and reflective subcomponents. That is, minoritized ethnic populations held strong negative emotions about participating in dementia research due to poor medical experiences often underpinned by experiences of systemic racism in healthcare, existing social stigmas around mental health/illness within their respective communities, and unfamiliarity with research. Researchers, too, rightfully perceived minoritized ethnic communities as being skeptical about dementia research, with a clear need for trust to be built. These insights support prior findings within similar populations (Black Americans) where experienced systemic racism acts as a salient barrier and the need for trust building is key for increasing research participation.^21^ Further, minoritized ethnic populations were consciously motivated to participate if they were able to see the practical consequences of doing so – supporting prior literature that it is important for dementia research to improve sharing of valuable healthy information with the community to promote participation interest.^40^

A significant strength of this study was its inclusion of diverse groups, including researchers, to gain a multiperspective understanding of the underrepresentation of minoritized ethnic groups in dementia research. Participants primarily came from the UK and mainland Europe, whose perspectives have been relatively underreported in this research area compared with North America. Additionally, more than half of the minoritized ethnic community member participants had not previously participated in research, allowing us to capture broader perspectives. This study also had limitations. While we focused on ethnicity, people are composed of multiple intersecting social characteristics (e.g., gender, sexual orientation, [dis]ability) that influence both their behavior and their experience of social positioning, marginalization, and inequity.^41^ From this intersectional perspective we note that the majority of participants in both the focus groups and researcher survey were women, which may have influenced the insights and recommendations reported here. Given that women have a higher risk of developing dementia,^2^ the overrepresentation of women in our sample may be appropriate. However, further efforts to engage men in this type of research, and more specifically to solicit views from men from minoritized ethnic backgrounds, is needed. Despite capturing multiple perspectives, only one person living with dementia participated in the project, and the views of people living with dementia may differ from those of family carers (who made up the majority of the focus groups). We also could not provide perspectives from those who are unable or unwilling to participate in research. Given that views expressed primarily came from participants in the UK and mainland Europe, we recommend that this work be replicated in other contexts to identify whether different perspectives based on culture or healthcare settings may emerge. Due to limited funding and staff resources, we only included participants who spoke fluent English, which meant that we ourselves fell prey to and perpetuated the barriers that we identified.

Our work dovetails with the well‐documented ethnicity‐based inequalities and inequities across healthcare (e.g., Irizar et al., LaVeist et al., Evandrou et al., Thompson et al.^42^, ^43^, ^44^, ^45^). While ultimately structural racism needs to be dismantled, increasing participation in research is one channel through which to improve healthcare equity – if research findings, which form the basis for clinical care, are derived from a limited segment of society, it cannot be assumed that those findings (and, therefore, the clinical care) would be appropriate and applicable to groups underrepresented in research.

### 4.1 Implications for practice

The study findings suggest two possible overarching behaviorally informed strategies that are likely to help reduce the current underrepresentation of minoritized ethnic populations in dementia research:

1. *Deploy targeted offline and online dementia research recruitment campaigns*
  Dementia research recruitment campaigns should involve (1) deployment in relevant locations both offline and online to maximize accessibility across age groups, (2) using relevant community languages to overcome language barriers, and (3) communicating in a culturally sensitive manner and utilizing relevant trustworthy personalities to increase trust and reduce social stigma. This might help increase minoritized ethnic populations' participation in dementia research. Specifically, it may do so by increasing knowledge about mental health/illness and routes to participation (psychological capability barrier), reducing existing mental illness stigmas (social opportunity barrier), and reducing existing negative emotions through trust building (automatic and reflective motivation).
2. *Fund, develop, and practice participatory dementia research programs*
  Systemic racism was identified as a transversal barrier affecting psychological skill deficits (e.g., knowledge about dementia and research opportunities) and negative emotions. Actions to dismantle institutional racism require coordination between multiple stakeholders, and funding is a key lever. It is essential that funding for dementia research be strategically utilized. Indeed, while funders increasingly encourage researchers to demonstrate how they intend to recruit participants from underrepresented backgrounds, this could be taken further. Funders and policymakers could require and support researchers to ensure ethnic minority representation in their projects.^46^ That is, funding should be focused on developing community‐based participatory dementia research programs.^47^ These programs should involve (1) hiring researchers from relevant minoritized ethnic backgrounds, (2) providing cultural competence training to existing researchers, (3) developing working and equitable relationships with relevant local minoritized ethnic background community members and institutions that span the full research cycle, (4) developing culturally adapted research materials, and (5) negotiating extended deadlines from grant funders to accommodate the longer required research timelines. These measures may help to increase participation of minoritized ethnic populations in dementia research by making the participation more accessible both in terms of location/time (physical opportunity barriers) and required language skills (psychological capability barrier), reduce existing mental illness stigmas, partly address systemic racial issues within healthcare systems (social opportunity barrier), reduce existing negative emotions through trust‐building, and make the research consequences more salient to the community (automatic and reflective motivation). It may also help to researchers increase their recruitment of minoritized ethnic populations in dementia research by providing them with relevant training (psychological capability barrier), essential research materials, and recruitment channels (physical opportunity barrier) and creating the overall necessary knowledge/skills to run such programs by diversifying dementia research teams (social opportunity barrier).

## CONFLICT OF INTEREST STATEMENT

All authors have nothing to disclose. Author disclosures are available in the Supporting Information.

## CONSENT STATEMENT

All participants in the study provided informed consent.

## Supporting information



Supporting Information

Supporting Information

Supporting Information

Supporting Information

Supporting Information
