# Supplementary material for: A multiperspective investigation of the underrepresentation of minoritized ethnic participants in dementia research and proposed strategies to improve inclusive recruitment practices
Source: Alzheimers Dement. 2025 Apr 6;21(4):e70129. doi: 10.1002/alz.70129 (PMC11973134; doi:10.1002/alz.70129)
Supplement: Supplementary file 2 — Supporting Information [file ALZ-21-e70129-s003.pdf]

Appendix A: Consolidated criteria for reporting qualitative studies (COREQ): 32-item checklist

Marchant et al. A multi-perspective investigation of under-representation of minoritised-ethnic participants in dementia research and proposed strategies to improve inclusive recruitment practices

No	Item	Guide questions/description	Location in manuscript
**Domain 1: Research team and reflexivity**			
Personal Characteristics			
1	Interviewer/facilitator	Which author/s conducted the interview or focus group?	Pg 8 Lns 208-209
2	Credentials	What were the researcher's credentials? *E.g. PhD, MD*	Pg 8 Lns 209-214
3	Occupation	What was their occupation at the time of the study?	Pg 8 Lns 209-214
4	Gender	Was the researcher male or female?	Pg 8 Lns 209-214
5	Experience and training	What experience or training did the researcher have?	Pg 8 Lns 209-214
Relationship with participants			
6	Relationship established	Was a relationship established prior to study commencement?	Pg 6 Lns 173-175
7	Participant knowledge of the interviewer	What did the participants know about the researcher? e.g. *personal goals, reasons for doing the research*	Pg 8 Lns 217-219
8	Interviewer characteristics	What characteristics were reported about the interviewer/facilitator? e.g. *Bias, assumptions, reasons and interests in the research topic*	Pg 8 Lns 217-219
**Domain 2: study design**			
Theoretical framework			
9	Methodological orientation and Theory	What methodological orientation was stated to underpin the study? *e.g. grounded theory, discourse analysis, ethnography, phenomenology, content analysis*	Pgs 9-10 Lns 237-280
Participant selection			
10	Sampling	How were participants selected? *e.g. purposive, convenience, consecutive, snowball*	Pg 6 Lns 171-175
11	Method of approach	How were participants approached? e.g. *face-to-face, telephone, mail, email*	Pg 6 Ln 175
12	Sample size	How many participants were in the study?	Pg 12 Ln 334<br>Pg 20 Ln 575
13	Non-participation	How many people refused to participate or dropped out? Reasons?	Pg 12 Lns 331-332
Setting			
14	Setting of data collection	Where was the data collected? e.g. *home, clinic, workplace*	Pg 7 Lns 196-197<br>Pg 8 Lns 215-216, 223-225
15	Presence of non-participants	Was anyone else present besides the participants and researchers?	n/a
16	Description of sample	What are the important characteristics of the sample? *e.g. demographic data, d*	Table 1, Table 2
Data collection			
17	Interview guide	Were questions, prompts, guides provided by the authors? Was it pilot tested?	Appendix B, Appendix C
18	Repeat interviews	Were repeat interviews carried out? If yes, how many?	n/a
19	Audio/visual recording	Did the research use audio or visual recording to collect the data?	Pg 8 Lns 223-225
20	Field notes	Were field notes made during and/or after the interview or focus group?	Pg 8 Lns 223-225
21	Duration	What was the duration of the interviews or focus group?	Pg 8 Ln 222
22	Data saturation	Was data saturation discussed?	Pg 10 Lns 281-293
23	Transcripts returned	Were transcripts returned to participants for comment and/or correction?	Pg 8 Lns 225-226
**Domain 3: analysis and findings**			
Data analysis			
24	Number of data coders	How many data coders coded the data?	Pg 11 Ln 294
25	Description of the coding tree	Did authors provide a description of the coding tree?	Pg 12 Lns 328-329<br>Appendix D
26	Derivation of themes	Were themes identified in advance or derived from the data?	Pg 11 Ln s 303-305
27	Software	What software, if applicable, was used to manage the data?	Pg 10 Ln 274
28	Participant checking	Did participants provide feedback on the findings?	Pg 11 Ln 306
Reporting			
29	Quotations presented	Were participant quotations presented to illustrate the themes / findings? Was each quotation identified? e.g. *participant number*	Pgs 12-27 Lns 339-769
30	Data and findings consistent	Was there consistency between the data presented and the findings?	Pgs 12-27 Lns 339-769
31	Clarity of major themes	Were major themes clearly presented in the findings?	Pgs 12-27 Lns 339-769
32	Clarity of minor themes	Is there a description of diverse cases or discussion of minor themes?	Pgs 12-27 Lns 339-769
