# Supplementary material for: A multiperspective investigation of the underrepresentation of minoritized ethnic participants in dementia research and proposed strategies to improve inclusive recruitment practices
Source: Alzheimers Dement. 2025 Apr 6;21(4):e70129. doi: 10.1002/alz.70129 (PMC11973134; doi:10.1002/alz.70129)
Supplement: Supplementary file 3 — Supporting Information [file ALZ-21-e70129-s002.docx]

**Appendix B – Focus Group Topic Guide**

**Open session**

- Facilitator introductions
- Reminders about participating (e.g. right to withdraw)
- Presentation about dementia with Q&A
- Describe purpose of the study

**Facilitated Discussion – Part 1**

- What does “research” mean to you? What is your understanding of research?
- Are you aware that you can take part in research?
- If you are aware that you can take part, then in what ways can you take part in research?
- If previously participated in research:
  - What was the research you took part in?
  - What was your experience like?
  - How did you get involved?
  - Why did you get involved, and what were your expectations from being involved in the research? Did the research meet your expectations? If not, how not? If so, how so?

**Break**

**Facilitated Discussion – Part 2**

- How would you like to hear about research that you can participate in?
- What would encourage you to participate in research?
- What do you think the barriers and facilitators to you taking part in research as a member of a community that has been under-served by researchers?
- Would you take part in research (again)? Why? Why not?
- In what ways do you think researchers could engage with your community to improve recruitment strategies so that people like yourselves can take part in research?

**Close session**

- Thank people for their time
- Reiterate what we’re going to do with this information
