# Supplementary material for: A multiperspective investigation of the underrepresentation of minoritized ethnic participants in dementia research and proposed strategies to improve inclusive recruitment practices
Source: Alzheimers Dement. 2025 Apr 6;21(4):e70129. doi: 10.1002/alz.70129 (PMC11973134; doi:10.1002/alz.70129)
Supplement: Supplementary file 4 — Supporting Information [file ALZ-21-e70129-s001.docx]

**Appendix C – Online Researcher Survey Questions**

**Please tell us about yourself**

1. **Which country/region are you based in (from where do you conduct the majority of your research):**

(dropdown list of countries)

1. **Which of the following best describes your gender?**

Man

Non-binary

Woman

Prefer to self-describe (please describe): Please describe your gender:________

Prefer not to say

1. **Which word or words would you use to describe your ethnic group/race? You can skip this question if you prefer not to say.**
2. **Do you consider yourself to be from a minoritized ethnic background? You can skip this question if you prefer not to say.**

Yes

No

Prefer not to say

1. **How old are you?**

20 - 24

25 - 29

30 - 34

35 - 39

40 - 44

45 - 49

50 - 54

55 - 59

60 - 64

65 and over

Prefer not to say

1. **Are you working at a university?**

Yes

University name:

Department:

No

Name of the institution where you work:

Department:

1. **What is your career stage:**

Undergraduate student

Graduate/post-graduate student

Post-doctoral researcher

Early-career researcher

Mid-career researcher

Senior researcher

Other: Please describe your career stage_____

1. **What is your job title:**
2. **What type of research do you conduct (tick all that apply):**

Basic science (e.g. cellular, molecular)

Biomarkers

Clinical trials

Drug Discovery/Development

Epidemiological

Ethics

Genetic

Health services

Neuroimaging

Neuropsychology

Public Health

Social care

Other: Please specify the type of research you conduct:

1. **Are you a member of any organisations/groups that explore diversity, disparities, inequality and/or ethnicity?**

Yes

Please list the names of these organisations/groups here:

No

**Please tell us about your current recruitment practices**

1. **In your most recent study that included human participants, did you explicitly study ethnicity and/or ethnic diversity?**

Yes

No

1. **In your most recent study that included human participants, did you record the ethnicity/ethnic group/race of participants in your sample?**

Yes

No: Why did you not record the ethnicity/ethnic group/race of your sample?

1. **In your most recent study that included human participants, did the ethnicity/ethnic group/race of your participants reflect the diversity of the population that you are studying?**

Yes

No

1. **Where and how did you recruit participants? (e.g. hospitals, doctors offices, the community, care homes...)**
2. **In your most recent study that included human participants, did you take additional steps in recruitment to increase the ethnic/racial diversity of participants?**

Yes: What did you do?

No

1. **In your latest publication that included human participants, did you report ethnicity/ethnic group/race of your sample?**

Yes

No: Why not report ethnicity/ethnic group/race of your sample?

1. **Is there anything else you want to tell us about your current recruitment practice?**

**Please tell us about your barriers and facilitators to recruiting more ethnically diverse participants**

1. **In your opinion, what barriers are there that restrict you from recruiting ethnically/racially diverse participants in research?**

(Please write a short paragraph describing what is difficult. You could also include things you have tried that didn't work and why you have not tried other things)

1. **In your opinion, what facilitators are there, or that could be put in place, to help you recruit more ethnically/racially diverse participants?**

(If the answer is money, what would you do with it? What else could your employer do? The research funders? Healthcare systems and providers?)
