# Supplementary figures and images for: A multiperspective investigation of the underrepresentation of minoritized ethnic participants in dementia research and proposed strategies to improve inclusive recruitment practices
Source: Alzheimers Dement. 2025 Apr 6;21(4):e70129. doi: 10.1002/alz.70129 (PMC11973134; doi:10.1002/alz.70129)

## Appendix D – Coding Tree

### 1. Focus Group Coding Tree

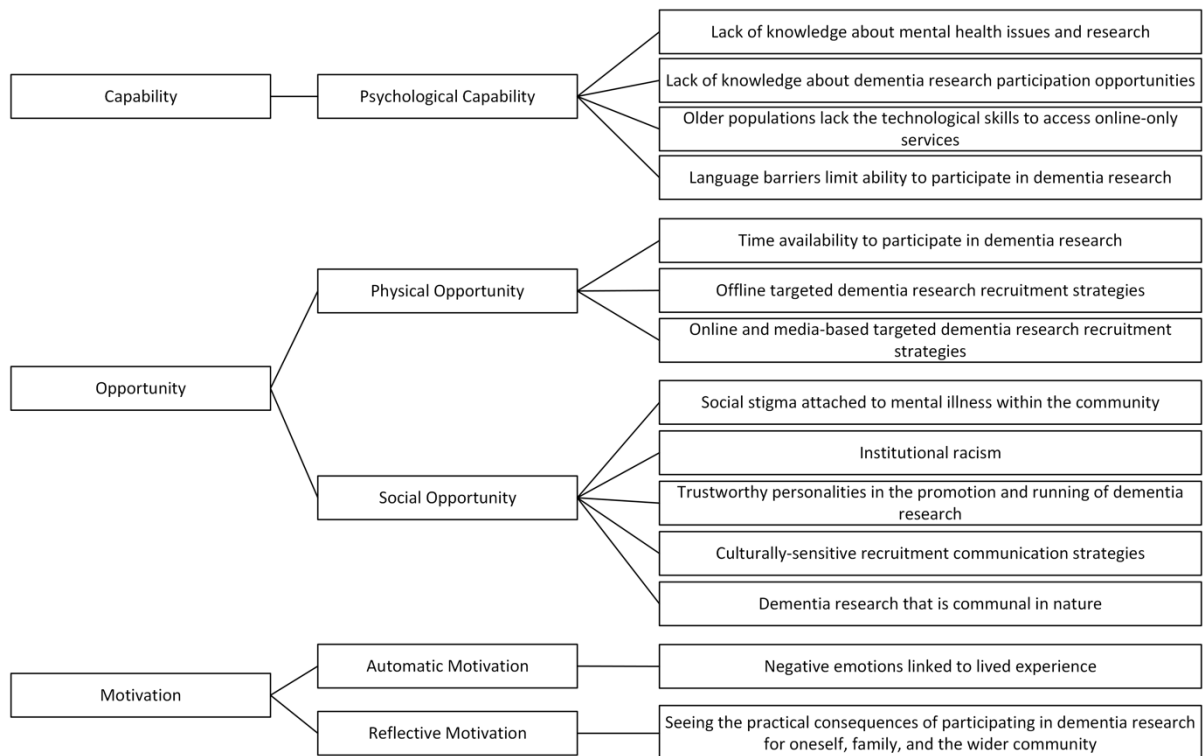

### 2. Researcher Survey Coding Tree

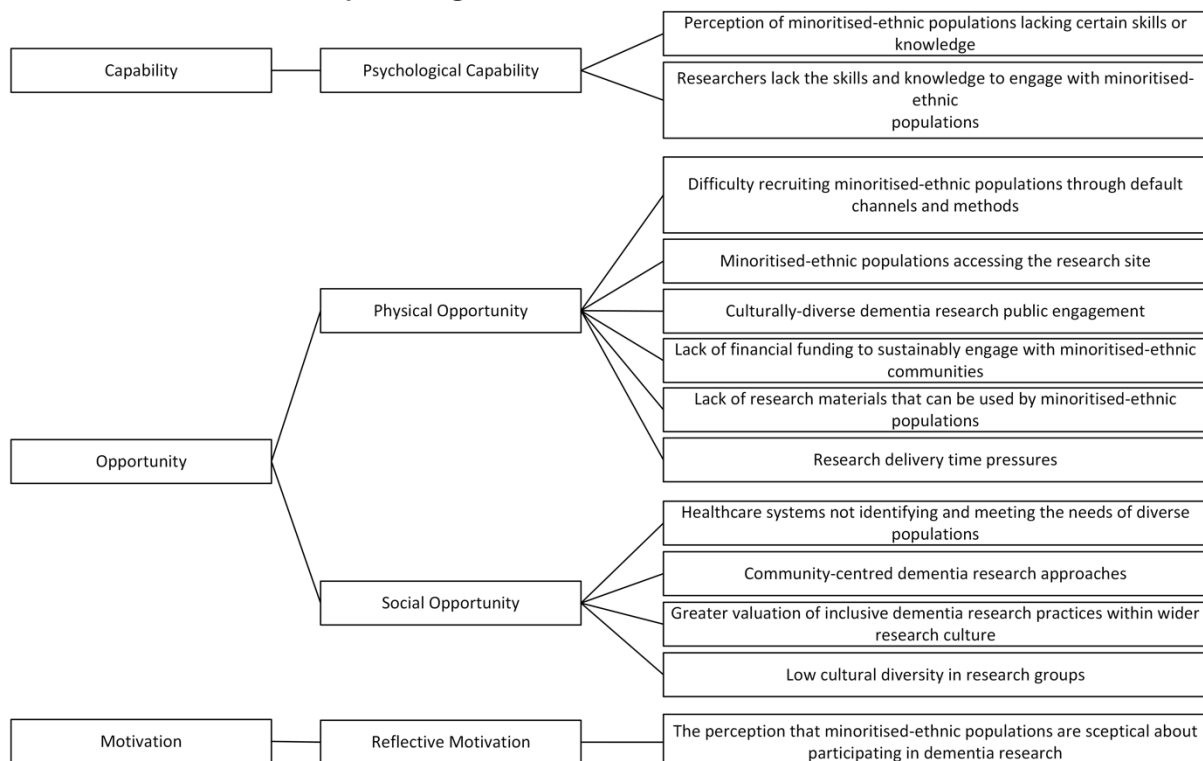

Supplement: Supplementary file 5 — Supporting Information [file ALZ-21-e70129-s004.pdf]
